# Efficacy of methylprednisolone for treatment of persistent vertigo

**DOI:** 10.1097/MD.0000000000017194

**Published:** 2019-09-20

**Authors:** Guo-rong Ding, Jian-ming Ni, Shan-jing Zhang, Yan-zhong Xie, Jun-fei Feng

**Affiliations:** a Department of Emergency; b Department of Respiratory Medicine, Hangzhou Fuyang Hospital of Traditional Chinese Medicine, Hangzhou, China.

**Keywords:** efficacy, methylprednisolone, persistent vertigo, safety

## Abstract

**Background:**

This study will systematically investigate the efficacy and safety of methylprednisolone for treatment of persistent vertigo (PV).

**Methods:**

All following electronic databases will be searched from inception to the June 30, 2019 without language restrictions: MEDILINE, EMBASE, Cochrane Library, Web of Science, and Chinese Biomedical Literature Database. All randomized controlled trials focusing on assessing the efficacy and safety of methylprednisolone for patients with PV will be fully considered for inclusion. Cochrane risk of bias tool will be used for assessing methodological quality, and RevMan 5.3 software (Cochrane Community, London, UK) will be utilized for statistical analysis.

**Results:**

This study will assess the efficacy and safety of methylprednisolone for PV via assessing primary outcome of vertigo, and secondary outcomes of somatization, depression, anxiety, health-related quality of life, and adverse events.

**Conclusion:**

This study will provide a high-quality evidence to judge whether methylprednisolone is an effective and safety therapy for patients with PV.

**Dissemination and ethics:**

No individual data will be utilized in this study, thus, it does not need ethical approval. The results of this study will be published at peer-reviewed journals.

**Systematic review registration:**

PROSPERO CRD42019138890.

## 1 Introduction

Persistent vertigo (PV) is one of the common complaints in the clinical practice.^[1,2]^ It occurs most commonly between the age of 50 and 70 years old.^[3,4]^ It has been estimated that its prevalence is 10.7 to 64 per 100,000 persons with a lifetime prevalence of 2.4%.^[5]^ Such disorder often manifests as patients’ heads or surrounding environments moving or spinning.^[6,7]^ In addition, it often accompanies dizziness, nausea and vomiting, tinnitus, and headache.^[8]^ Many factors can result in such condition, including labyrinthitis, vestibular neuronitis, cholesteatoma, Menière's disease, and so on.^[9,10]^

Numerous studies have reported to treat PV, such as videonystamography, prednisone, zolmitriptan, balance vestibular rehabilitation therapy, betahistine dihydrochloride, home-based exercise program, acupuncture, moxibustion, and methylprednisolone, especially for methylprednisolone.^[11–19]^ Although several studies have investigated the efficacy of methylprednisolone for patients with PV, no systematic review has systematically assessed its efficacy with clear conclusion.^[19–23]^ Therefore, this study will evaluate the efficacy and safety of methylprednisolone for PV.

## 2 Methods

### 2.1 Objective

This study aims to investigate the efficacy and safety of methylprednisolone for patients with PV.

### 2.2 Study registration

This study has been registered on PROSPERO with CRD42019138890. It has reported based on the guidelines of the Preferred Reporting Items for Systematic Reviews and Meta-Analysis (PRISRMA) Protocol statement.

### 2.3 Inclusion criteria for study selection

#### 2.3.1 Types of studies

All randomized controlled trials (RCTs) of methylprednisolone for patients with PV will be eligible for inclusion. However, non-clinical trials, and non-RCTs will be excluded.

#### 2.3.2 Types of participants

People of any age, race, and sex with PV will be considered for inclusion in this study.

#### 2.3.3 Type of interventions

The patients in the experimental group must be treated with methylprednisolone.

The patients in the control group can receive any treatments, but not the methylprednisolone.

#### 2.3.4 Type of outcome measurements

The primary outcome is vertigo, as measured by vertigo-related handicap and related scales. The secondary outcomes include somatization, as measured Patient Health Questionnaire 15-Item Somatic Symptom Severity Scale; depression, as measured by dizziness handicap inventory; anxiety, as measured by Beck Anxiety Inventory; health-related quality of life, as measured by 36-Item Short Form Survey; and adverse events.

### 2.4 Search methods for the identification of studies

We will seek all RCTs which evaluate the efficacy and safety of methylprednisolone for the treatment of PV.

#### 2.4.1 Electronic searches

We will search the following electronic databases for RCTs which assess the efficacy and safety of methylprednisolone for PV from inception to the June 30, 2019 without language limitations: MEDILINE, EMBASE, Cochrane Library, Web of Science, and Chinese Biomedical Literature Database. We will apply detailed strategy for electronic database MEDLINE and is showed in Table 1. The equivalent detailed strategies will also be applied to the other electronic databases.

**Table 1:**
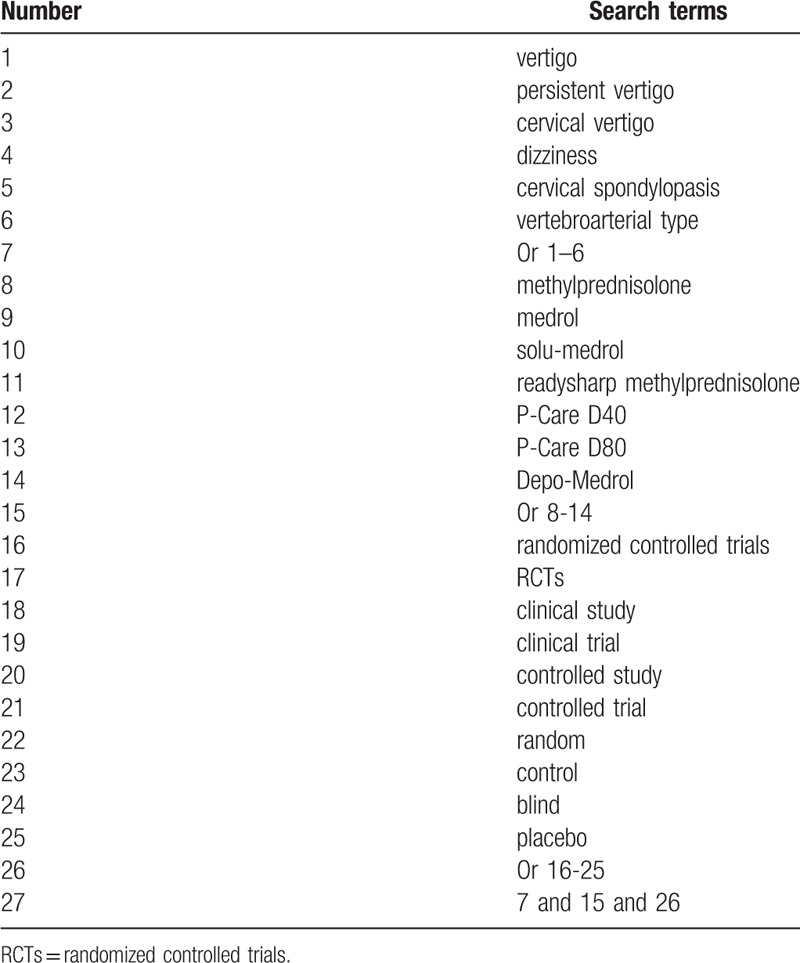
Search strategy for MEDLINE.

#### 2.4.2 Search for other resources

In addition, we will also search other bibliographic literature records, such as websites of clinical registry, dissertations, and reference lists of included studies to avoid missing any potential studies.

### 2.5 Data collection and analysis

#### 2.5.1 Study selection

Two researchers will independently assess titles and abstracts of literature records identified by the search strategy against the eligibility criteria for inclusion. Then, we will exclude irrelevant studies. After that, full versions of potential relevant studies will be screened against all eligibility criteria. Any differences in opinion will be solved by a third researcher through discussion. The process of all study selection will be presented in the Preferred Reporting Items for Systematic Reviews and Meta-Analyses (PRISMA) flow chart.

#### 2.5.2 Data extraction and management

Two researchers will independently extract data from the eligible studies using a data extraction form. Any differences between 2 researchers will be settled down by discussion with a third researcher.

We will extract the following information specifically:

- Study characteristics: title, authors, year of publication, location, study design;
- Patient characteristics: race, sex, diagnostic criteria, eligibility criteria;
- Study design: sample size, randomization, allocation concealment, blinding;
- Treatment and control details: types of interventions, dosage, frequency;
- Outcome details: all outcome measurements, safety.

#### 2.5.3 Missing data management

If there is evidence of missing or insufficient data, we will contact primary authors to request those data. If we cannot get those data, we will address the potential impact of those missing data in the discussion section.

#### 2.5.4 Methodological quality assessment

Two researchers will independently evaluate the included studies using Cochrane risk of bias tool. A third researcher will help to solve any different opinions between 2 researchers regarding the methodological quality assessment. This tool addresses 7 specific domains, and each domain will be further judged as high risk of bias, unclear risk of bias, and low risk of bias.

### 2.6 Statistical analysis

#### 2.6.1 Measurement of treatment effect

For continuous outcome data, we will calculate them as mean difference or standardized mean difference with 95% confidence intervals (CIs). For dichotomous outcome data, we will calculate them as odd ratio or risk ratio with 95% CIs.

#### 2.6.2 Assessment of heterogeneity

We will explore clinical heterogeneity using *I*^2^ statistic among eligible studies. Value of *I*^2^ ≤ 50% indicate satisfied heterogeneity, while values of *I*^*2*^ > 50% exert substantial heterogeneity.

#### 2.6.3 Data synthesis

If *I*^2^ ≤ 50%, we will use fixed-effect model, and data will be pooled and meta-analysis will be conducted. On the other hand, if *I*^*2*^ > 50%, random-effect model will be used, and a subgroup analysis will be carried out. If there is still substantial heterogeneity, data will not be pooled, and meta-analysis will not be performed. However, we will report results as narrative summary description.

#### 2.6.4 Subgroup analysis

If sufficient data are available, we will perform subgroup analysis in accordance with the different study settings, treatments, comparators, and outcomes.

#### 2.6.5 Sensitivity analysis

We will perform sensitivity analysis to check the robustness of outcome results by removing studies of the lowest methodological quality.

#### 2.6.6 Reporting bias

If sufficient eligible studies are included, we will perform funnel plot and Egger test to identify if there is reporting bias.

## 3 Discussion

Currently, various managements for PV can alleviate symptoms to a certain extent and decrease onset of PV,^[11–19]^ but their efficacy is still limited. Previous clinical studies reported that methylprednisolone can effectively treat such disorder.^[19–23]^ However, no study has systematically investigated the efficacy and safety of methylprednisolone for the treatment of patients with PV. This study will comprehensively search literature records and will systematically explore and provide most recent evidence on the efficacy and safety of methylprednisolone for patients with PV. Its findings may provide helpful evidence for clinician and patients.

## Author contributions

**Conceptualization:** Guo-rong Ding, Shan-jing Zhang, Yan-zhong Xie, Jun-fei Feng.

**Data curation:** Guo-rong Ding, Jian-ming Ni, Yan-zhong Xie, Jun-fei Feng.

**Formal analysis:** Jian-ming Ni, Shan-jing Zhang.

**Funding acquisition:** Guo-rong Ding.

**Investigation:** Guo-rong Ding, Yan-zhong Xie, Jun-fei Feng.

**Methodology:** Jian-ming Ni, Shan-jing Zhang.

**Project administration:** Guo-rong Ding, Yan-zhong Xie, Jun-fei Feng.

**Resources:** Jian-ming Ni, Shan-jing Zhang.

**Software:** Guo-rong Ding, Jian-ming Ni, Shan-jing Zhang.

**Supervision:** Yan-zhong Xie, Jun-fei Feng.

**Validation:** Guo-rong Ding, Shan-jing Zhang, Yan-zhong Xie, Jun-fei Feng.

**Visualization:** Guo-rong Ding, Jian-ming Ni, Yan-zhong Xie, Jun-fei Feng.

**Writing – original draft:** Guo-rong Ding, Jian-ming Ni, Shan-jing Zhang, Yan-zhong Xie, Jun-fei Feng.

**Writing – review & editing:** Guo-rong Ding, Jian-ming Ni, Shan-jing Zhang, Yan-zhong Xie, Jun-fei Feng.
